# Platelet Aggregation and Mental Stress Induced Myocardial Ischemia: Results from the REMIT Study

**DOI:** 10.1016/j.ahj.2014.12.002

**Published:** 2014-12-17

**Authors:** Wei Jiang, Stephen H. Boyle, Thomas L. Ortel, Zainab Samad, Eric J. Velazquez, Robert W. Harrison, Jennifer Wilson, Cynthia Kuhn, Redford B. Williams, Christopher M. O’Connor, Richard C. Becker

**Affiliations:** a Department of Psychiatry, Duke University Medical Center, Durham, NC, USA; b Department of Medicine, Duke University Medical Center, Durham, NC, USA; c Duke Clinical Research Institute, Durham, NC; d Department of Pharmacology & Cancer Biology, Duke University, Durham, NC; e Department of Medicine, University of Cincinnati, Cincinnati, OH

**Keywords:** Mental stress-induced myocardial ischemia, mental stress, platelet aggregation

## Abstract

**BACKGROUND:**

Mental stress-induced myocardial ischemia (MSIMI) is common in patients with ischemic heart disease (IHD) and associated with a poorer cardiovascular prognosis. Platelet hyperactivity is an important factor in acute coronary syndrome. This study examined associations between MSIMI and resting and mental stress-induced platelet activity.

**METHODS:**

Eligible patients with clinically stable IHD underwent a battery of 3 mental stress tests during the recruitment phase of REMIT (Responses of Myocardial Ischemia to Escitalopram Treatment) study. MSIMI was assessed by echocardiography and electrocardiography. *Ex vivo* platelet aggregation in response to ADP, epinephrine, collagen, serotonin, and combinations of serotonin plus ADP, epinephrine, and collagen were evaluated as was platelet serotonin transporter expression.

**RESULTS:**

Of the 270 participants who completed mental stress testing, and had both resting and post-stress platelet aggregation evaluation, 43.33% (N=117) met criteria for MSIMI and 18.15% (N=49) had normal left ventricular response to stress (NLVR). The MSIMI group, relative to the NLVR groups, demonstrated heightened mental stress-induced aggregation responses, as measured by area under the curve, to collagen 10 μM (6.95[5.54] vs. −14.23[8.75].; p=0.045), epinephrine 10 μM (12.84[4.84] vs. −6.40[7.61].; p=0.037) and to serotonin 10 μM plus ADP 1 μM (6.64[5.29] vs. −27.34[8.34]; p < .001). The resting platelet aggregation and serotonin transporter expression, however, were not different between the two groups.

**CONCLUSIONS:**

These findings suggest that the dynamic change of platelet aggregation caused by mental stress may underlie MSIMI. While the importance of these findings requires additional investigation, they raise concern given the recognized relationship between mental stress-induced platelet hyperactivity and cardiovascular events in patients with IHD.

## INTROUCTION

Mental stress-induced myocardial ischemia (MSIMI) occurs commonly in patients with clinically stable ischemic heart disease (IHD) (1,2). Our recent study demonstrated MSIMI is more common than exercise stress-induced ischemia (43.5 vs. 33.8%, p= 0.003) in patients with clinically stable IHD. Occurrence of MSIMI in IHD patients is a risk factor for acute myocardial infarction (MI), unstable angina, coronary revascularization, and death(3,4). MSIMI is recognized as an intermediate and integrated biomarker linking psychosocial variables to IHD, and may be a surrogate endpoint to evaluate effective therapeutics attempting to modify the negative connection between psychosocial variables and IHD (5).

Platelets play a major role in the pathogenesis of atherosclerosis and coronary thrombosis (6–8) as many studies have demonstrated the importance of increased platelet aggregation in coronary artery atherosclerosis and thrombotic disorders, such as myocardial infarction (MI), stroke and cardiovascular death (9–11). With increased evidence supporting the role of psychosocial risk factors in IHD development and progression, many studies have examined the effect of acute emotional or mental stress on platelet activities in healthy individuals and patients with angina and/or IHD. Results of these studies have generally demonstrated that mental stress elevates platelet activities (12,13) Alterations in platelet aggregation due to mental stress may be one mechanism underlying MSIMI but no study has investigated this possibility.

In the current study, we examined the association of resting and mental stress-induced platelet aggregation with MSIMI status using data collected from the baseline screening of the REMIT (Responses of Myocardial Ischemia to Escitalopram Treatment) study. We also examined the relationship of platelet serotonin transporter expression and MSIMI status. Our primary hypothesis was, compared to patients who had normal left ventricular response (NVLR) to mental stress, patients who developed MSIMI would demonstrate higher platelet aggregation in response to physiologic agonists at rest and after mental stress testing.

## METHODS

The REMIT study applied mental stress testing to patients with clinically stable IHD. Patients who developed MSIMI were then randomized to escitalopram or placebo in a double-blind fashion for 6-weeks. The study was conducted at the Duke University Health System in the United States. The study protocol was reviewed and approved by the Duke Institutional Review Board, and all participants provided voluntary written informed consent. Detailed description of the REMIT study methodology and the characteristics of MSIMI have been published (2,5,14).

### Population

Clinically stable adult patients aged ≥21 years with documented coronary artery disease were eligible for participation (14). Exclusion criteria included significant cognitive impairment, life-threatening comorbidity (estimated 50% mortality within 1 year), active suicidal ideation, warfarin usage, and psychiatric conditions precluding SSRI use. The present study examined those patients who underwent baseline mental stress testing for the evaluation of myocardial ischemia, who also provided a blood sample for platelet aggregation tests.

### Mental Stress Testing and Mental Stress Induced Myocardial Ischemia

Stress tests were conducted at the Duke Cardiac Diagnostic Unit in the morning following 24–48 hours withholding of beta-blockers.

Participants undertook a 20-minute calibration-rest period, and then were asked to complete a series of 3 mental stress tasks in a fixe sequence. Each mental task lasted for 3 minutes. A brief description of these tasks is at the followings. (1) Mental arithmetic: patients were asked to perform a series of serial subtractions beginning at a given number which were different for each repeated test and chosen by the tester from a fixed list of various numbers, with encouragement to perform calculations as quickly and accurately as possible; (2) Public speaking with anger recall: patients were asked to give a brief speech on a recent situation in which they experienced anger or upset to an audience of 2–3 observers after 1 minute of preparation. Participants were instructed to have the speech well organized with clearly defined issue and concise content, as well as the speech would be evaluated. If they run out of what to say, the research staff would prompt them with questions to elicit more content until the 3-minute was up; (3) Mirror trace: patients were asked to outline, as quickly and accurately as possible, a star from its reflection in a mirror. The device of the mirror trace beeps and records errors participants made during the testing.

Echocardiography and electrocardiography (ECG) were used to assess MSIMI. Wall motion assessments were determined from systole from 1 cardiac cycle at a frame rate of 30–40 frames/second using the 16-segment model recommended by the American Society of Echocardiography. Left ventricular ejection fraction (LVEF) was calculated from a 3–5 beat loop. These assessments were blinded from the stress tests. To minimize variation and temporal drift and to enhance reliability, all echo images of participants were batch read by 2 experienced cardiologists (E.V. and Z.S.).

### Definition of the Study Groups

The MSIMI group was comprised of patients with MSIMI, defined by presence of ≥1 ischemic markers: compared to rest, (1) any development or worsening of wall motion; (2) reduction of LVEF ≥8%; and/or (3) deviation of ST-segment in ≥2 leads lasting for ≥3 consecutive beats, occurring during ≥1 of the 3 mental stress tasks(5,15). The NLVR group consisted of patients who did not have MSIMI, and whose mental stress induced LVEF changes were zero or greater from baseline resting LVEF to all three mental tasks. Patients who did not meet MSIMI criteria but had mental stress induced LVEF reduction from baseline resting in response to at least one mental task comprised the intermediate group.

### Measurement of Platelet Activities

#### Sample Preparation

Upon arrival to the laboratory, a 19-gauge butterfly needle was inserted in the antecubital vein of the arm opposite from the arm where the blood pressure cuff was placed to allow for acquisition of blood samples with minimum disturbance. Subjects then rested quietly for 20 minutes to allow hormones and other biochemicals to return to normal resting. Following the rest period, participants underwent the three mental stress tasks in a fixed sequence: Mental arithmetic, Mirror trace, and Anger recall. A rest period of 6 minutes was applied after each stress test. Blood samples were collected after the 20-minute resting period prior to preparation of beginning mental stress testing and at the end of the third mental stress test that was approximately 28 minutes after the initiation of the first mental task. All blood samples were collected in mornings to minimize the circadian variation in platelet activity. The blood samples were collected into vacutainer tubes containing 3.8% sodium citrate solution (9:1) after the first 4 ml was discarded with collection strategies designed to minimize platelet aggregation in the aliquot. Blood samples were then transferred immediately to the lab in room temperature and divided for sample preparation and platelet serotonin uptake assay. Platelet-rich plasma (PRP) was prepared from whole blood by centrifugation at 180g for 15 minutes, and platelet-poor plasma (PPP) was prepared by centrifugation at 2000g for 10 minutes. Platelet aggregation and serotonin uptake were assessed immediately, and platelet membranes stored frozen until the binding assay was conducted.

#### Platelet Aggregation

Platelet aggregation assays were performed in the Duke Hemostasis and Thrombosis Core under the direction of a co-author of the study (T.L.O.) and were completed within one hour of each blood draw. Light transmittance aggregometry (LTA) was performed according to the method of Born (16–18). Platelet activation *in vivo* generally involves a combination of agonists. Because serotonergic and adrenergic stimuli as well as collagen and ADP affect platelet activity *in vivo*, an investigation of the biological response to multiple stimuli is likely to identify physiological effects that have implications for the pathogenesis of thrombosis in stress (19). Thus, we tested *ex vivo* platelet aggregation responses to different agonists. Aggregation triggered by individual epinephrine (2, 5 and 10μM), serotonin (10μM), collagen (2, 5 and 10 μg/ml) and ADP (1, 2, and 5μM), as well as serotonin (10 μg/ml) combined with ADP(1 μM), epinephrine (2 μM), or collagen (2 μg/ml) in samples collected at rest and after mental stress testing were evaluated. We chose the area under the LTA curve (AUC) as the primary measure of aggregation because the AUC captures several features of the aggregometry measurement that are sensitive to the effects of aspirin: slope (20,21) maximal aggregation (22), and final aggregation (23). To standardize measurements across individuals and visits we fixed the testing duration at 6 min for all agonists and serotonin. All samples were processed and the platelet aggregation studies were performed by the same experienced technician using one instrument and an identical lot of agonists.

#### Platelet Serotonin Transporter Expression

Platelet serotonin transporter function was assessed with [3H] serotonin uptake, and transporter expression was quantitated with [3H]-paroxetine binding as described by Nemeroff (24,25) and modified by Slotkin et al. (19,26). Only resting samples were evaluated. PRP was obtained by centrifugation of whole blood at 100 g for 30 min. The PRP was centrifuged (39,000 × g for 10 min at 4°C) and lysed by suspension in 5 mM Tris buffer (pH 7.5) containing 5 mM EDTA, pH 7.5, sedimented at 39,000 g suspended in 70 mM Tris (pH 7.5), resedimented, and finally resuspended in the assay buffer (50 mM Tris (pH 7.5) containing 120 mM NaCl and 5mM KCl). Aliquots were taken for protein determination and the suspension frozen at −70°C until assay at a concentration of 1 mg protein/ml. Binding of [3H]-paroxetine to platelet membranes was accomplished by incubating aliquots of platelet membranes in six different concentrations of [3H]-paroxetine (25, 50, 100, 250, 500, 1,000 pM) in triplicate using 100 μg of platelet protein/tube in a final volume of 250 μl assay buffer. Tubes were incubated on ice for 60 min, after which 5 ml of ice-cold buffer were added and labelled membranes harvested by vacuum filtration on Whatman GF/C glass fiber papers pre-soaked in 0.05% polyethyleneimine. Filters were washed three times with 5 ml of ice-cold buffer, and filter papers counted by liquid scintillation spectrometry using a non-toluene based fluor (Safety Solv). Nonlinear regression algorithm for sigmoid curves with Prism 3.0 (Graphpad, San Diego, CA, USA) was used to determine serotonin transporter maximal binding capacity (Bmax, fmol/mg protein), dissociation constant or binding affinity (Kd100, nM), and platelet serotonin uptake rate (Vmax, fmol/107 platelets per 5 minutes). Non-specific binding was determined as the binding in the presence of 0.22 mM serotonin.

### Psychological Measurements

Severity of depressive symptoms was assessed by the Beck Depression Inventory II (BDI) (27) and the Center for Epidemiologic Studies Depression Scale (CESD) (28). The BDI consists of 21 items that cover emotional, behavioral, and somatic symptoms. The CESD is a 20-item, questionnaire in which patients report on the frequency of depressive symptoms experienced in the past 2 weeks using a 4-point Likert scale. Anxiety was assessed using the 40-item Spielberger Anxiety Scale(29) which measures state (SSA) and trait (STA) manifestations of anxiety.. Level of stress was measured via the 10-item Perceived Stress Scale (PSS) that measures the degree to which situations in one’s life are appraised as stressful(30). All were self-administered.

### Statistical Analysis

The primary focus of our analysis was to document differences between the MSIMI and NLVR groups. We excluded the intermediate group from the primary analysis as it is currently unclear whether a mental stress-induced LVEF reduction < 8 is a definitive sign of ischemia. We used T-tests and chi-square analysis to test for bivariate associations between the MSIMI status (i.e. MSIMI vs NLVR) and the demographic and clinical variables of interest.

As previously described, platelet aggregation was measured in response to several different concentrations of the individual agonists. We have observed that platelet aggregation in response to different concentrations of the same agonist to be highly correlated and that the greatest aggregations were induced by the highest concentrations of each agonist. Therefore, the focus of our analysis was on the platelet aggregation in response to the highest concentration of each agonist. We felt this strategy was adequate for testing our hypotheses while allowing for a simpler and more concise presentation of the data.

We used analysis of covariance (ANCOVA) and multivariate logistic regression to examine associations of resting platelet aggregation and platelet aggregation change scores (post stress values – pre-stress values) to MSIMI status. These models included sex, resting LVEF, age, smoking status, BDI scores, and resting stimulated platelet aggregation (only for analyses of platelet aggregation change scores) as covariates. A number of patients (N =12) were missing resting LVEF values, therefore, we used mean imputed resting LVEF values in all analyses. Separate ANCOVAs were fitted for the AUC of each stimulated platelet aggregation to agonist (i.e., ADP, epinephrine, collagen, serotonin, serotonin + ADP, serotonin + epinephrine, and serotonin + collagen). Platelet aggregation responses to different agonists tend to be moderately intercorrelated suggesting that there is substantial redundancy among the platelet aggregation variables used in this study. Therefore, we used principal component analysis (PCA) to construct composite scores comprised of linear combinations of the stress-induced platelet aggregation variables. For the purpose of this paper we performed two PCAs, one that included baseline adjusted stress-induced platelet aggregation to the individual agonists (i.e. epinephrine, ADP, and collagen) and one that included baseline adjusted stress-induced platelet aggregation to the combination of serotonin with each of the three individual agonists. The composite scores were also examined as correlates of MSIMI status with our ANCOVA and multivariate logistic regression models. An advantage of the use of the composite scores in our analysis is that they provide a test of the central hypothesis of our study (i.e. is stress-induced platelet aggregation associated with MSIMI) while minimizing the effect of multiple tests. Composite scores are expressed as z-scores and have a mean of 0 and a standard deviation of 1.

We also examined associations between several secondary variables of interest and MSIMI status. These variables included stress induced cardiovascular (SBP, DBP, HR, LVEF, and wall motion score index [WMSI]) and emotional responses (sadness, tension, frustration, calm, and in-control) and measures of serotonin receptor transporter expression (Bmax, Kd100, Vmax). As with the analysis of the platelet aggregation variables, these models included the following covariates: sex, age, BDI scores, smoking status, resting LVEF, and the appropriate resting cardiovascular or state emotion variable for analyses of mental stress-induced responses.

Area under the receiver operating characteristic curves (ROC) were used to evaluate how well the platelet aggregation reactivity variables predicted MSIMI. For the purpose of this analysis, we focused on platelet reactivity variables that showed statistically significant relationships to the MSIMI variable in the primary analysis. The ROC curves were also used to see if we could identify the optimal cut point of the adjusted platelet aggregation reactivity variables for predicting MSIMI status. All analyses were performed using SAS statistical software, version 9.1 (SAS Institute Inc, Cary, NC) and a p-value of .05 was used to determine significance.

## RESULTS

### Baseline Demographic and Clinical Characteristics

Of the 335 clinically stable CHD patients who provided consent and underwent the baseline assessments of REMIT study (2), 270 had complete data from the mental stress testing and resting/post stress platelet aggregation tests. One patient was missing data for a key covariate, resulting in an available N of 269 for adjusted analyses. Baseline demographic and clinical characteristics of the study population, divided into MSIMI, NLVR, and Intermediate groups, are summarized in Table 1. Consistent with data that has been previously presented (2), the majority of these variables were not significantly different between MSIMI and NLVR groups. As previously reported, MSIMI was significantly more prevalent in female than male patients (19.0% vs. 4.08%, p=0.011) (Table 1). The MSIMI group appeared to have greater levels of distress as indicated by the higher scores on the psychological measures and state negative affect and the lower scores on the measures of state positive affect, however none of these differences were statistically significant (Table 1 and Table 2). Table 2 presents cardiovascular function measurements collected during the baseline rest period of the mental stress protocol. Compared to the NLVR group, the MSIMI group had higher resting WMSI scores (1.15 vs. 1.29, p = .044) and similar resting LVEF values (55.51 vs. 55.27%, p = .90) (Table 2).

### Platelet Aggregation

**Figure 1:**
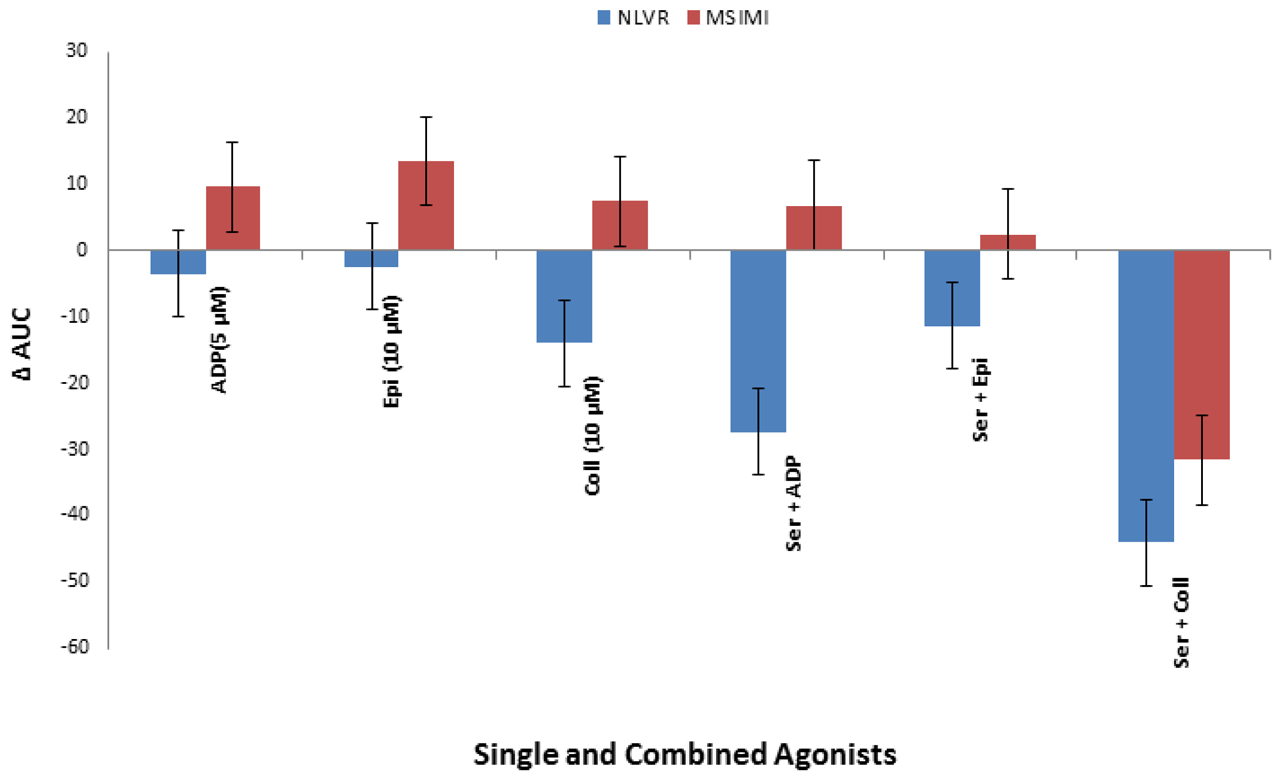
Mental Stress-Induced Platelet Aggregations between MSIMI (mental-stress induced myocardial ischemia) and NLVR (normal left ventricular response) groups Epi = Epinephrine, Coll = Collagen, and Ser = Serotonin Note: Positive AUC values indicate an increase in platelet aggregation in response to mental stress and negative AUC values indicate a decrease in platelet aggregation in response to mental stress.

Resting platelet aggregation to each agonist was similar in the MSIMI and NLVR groups (Table 2). In contrast, there were differences in post-stress platelet responses between the MSIMI and NLVR groups (Table 3). On average, mental stress- induced platelet aggregation to each the agonist, except to serotonin plus collagen, increased in the MSIMI group and decreased in the NLVR group compared to their resting measurements (Figure 1). There were significant associations between MSIMI status and stress- induced aggregation to collagen 10 μM (−14.23[8.75] vs. 6.95[5.54], p = .045), epinephrine 10 μM (−6.40[7.61] vs. 12.84[4.84], p = .037) and to serotonin + ADP (−27.24[8.34] vs. 6.64[5.29], p < .001), (Table 3). To control for possible effects of antiplatelet medication usage on the platelet aggregation measures, we refitted the collagen 10μM, epinephrine 10 μM, and serotonin + ADP models including the variable of platelet P2Y_12_ receptor blocker (PPRB) usage as a covariate. Control for PPRB usage had little effect on the association of between MSIMI status and collagen 10 μM (p=.052), epinephrine 10 μM (p = .036) and serotonin + ADP (p < .001) triggered mental stress-induced platelet aggregation. Multivariate logistic regression analysis also showed that those platelet aggregation variables were significant predictors of MSIMI status (Table 4). The corresponding odds ratios OR and 95% confidence intervals (CI) were 1.006(95%CI = 1.000 – 1.012), p = .054 for collagen 10 μM, 1.009 (95%CI = 1.000 – 1.017), p = .054 for epinephrine 10 μM, and 1.012(95%CI= 1.005–1.02), p = .001 for serotonin + ADP.

Further analysis of the entire study (N=270) population revealed that mental stress induced platelet aggregation to serotonin +ADP was significantly higher in patients with MSIMI than in the remainder of patients (i.e. the combination of the NLVR and intermediate groups, MSIMI-No: −14.33[4.68] vs. MSIMI-Yes: 5.91[5.35]), p = .005). A correlation analysis of this sample revealed significant correlations between the mental stress-induced platelet aggregation to serotonin + ADP and mental stress-induced WMSI (r =.15, p = .014) and LVEF changes (r = −.17, p = .007). None of the other correlations were statistically significant.

The PCA of the individual agonist variables yielded a single component (eigenvalue = 1.59) accounting for 53% of the variance. The PCA of the combined agonists also yielded a single component (eigenvalue = 2.01) accounting for 67% of the variance. The high percentage of variance accounted for by these two components suggests that the composite scores derived from them are capturing much of the variance of the individual platelet aggregation variables that comprised each component. ANCOVAs revealed significant effects for both the individual agonist component (p = .030) and the combined agonist component (p= .019). In both cases, the MSIMI group demonstrated higher component scores indicating higher stress-induced pa reactivity relative to those in the NLVR group. Both the individual agonist (OR = 1.57(95%CI = 1.05 – 2.36), p = .028) and serotonin +agonist (OR = 1.59(95%CI = 1.07 – 2.37), p = .022) factor scores were significant predictors of MSIMI status in multivariate logistic regression (Table 4).

### Platelet Serotonin Transporter Expression

The measurements of platelet serotonin transporter expression, i.e., Vmax, Bmax and Kd100 were not different between the groups (Table 2).

### Mental stress Induced Cardiovascular and Emotional Responses

MSIMI patients had slightly lower SBP, DBP, and HR reactions to the mental stress testing compared to NLVR patients, but none of the differences was statistically significant. Emotional responses in the MSIMI group tended to be greater in the negative direction compared to the NLVR group, but the differences between the groups were not statistically significant (Table 3).

### Predictability of Mental stress-Induced Platelet Aggregations for MSIMI

**Figure 2:**
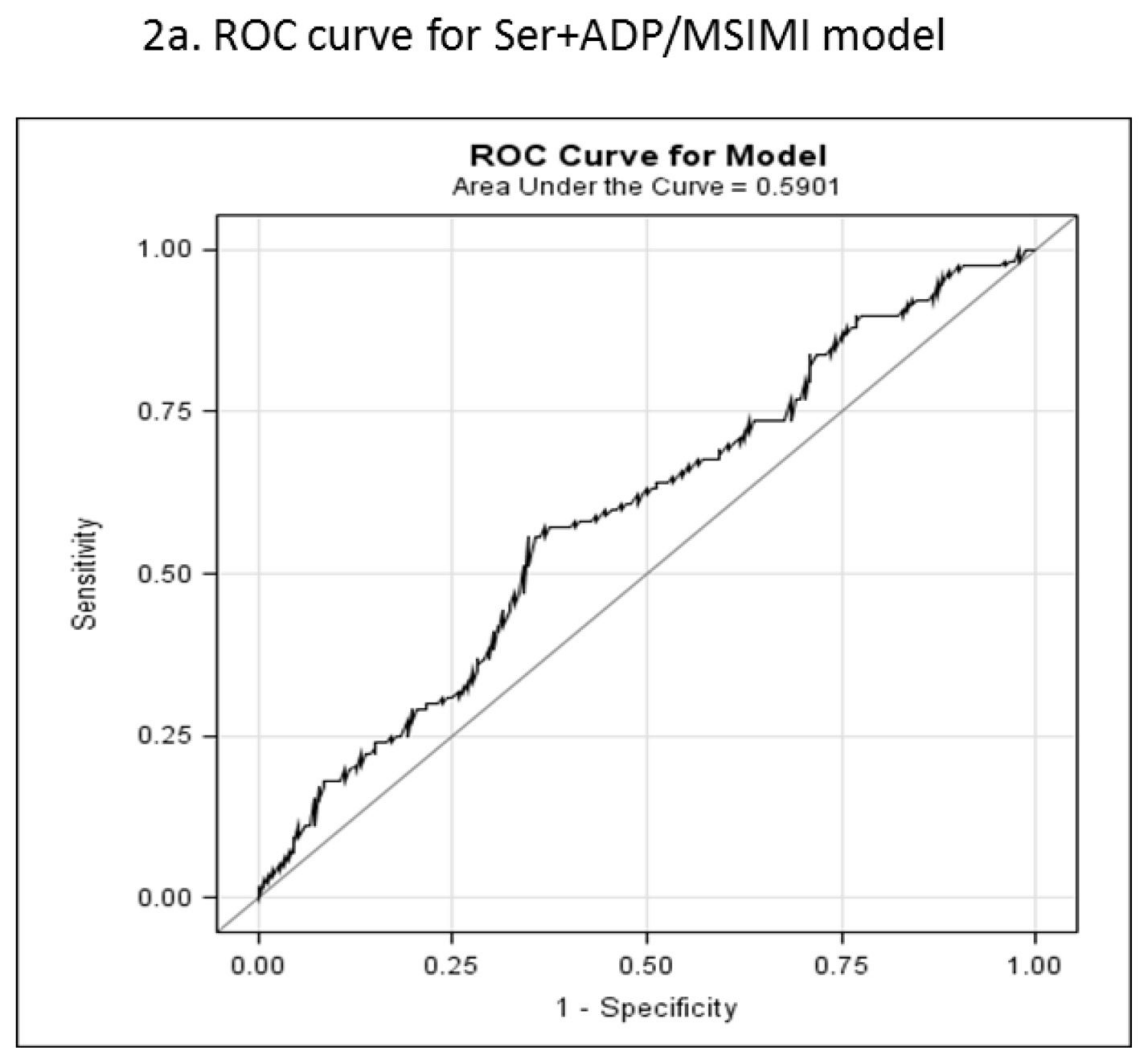

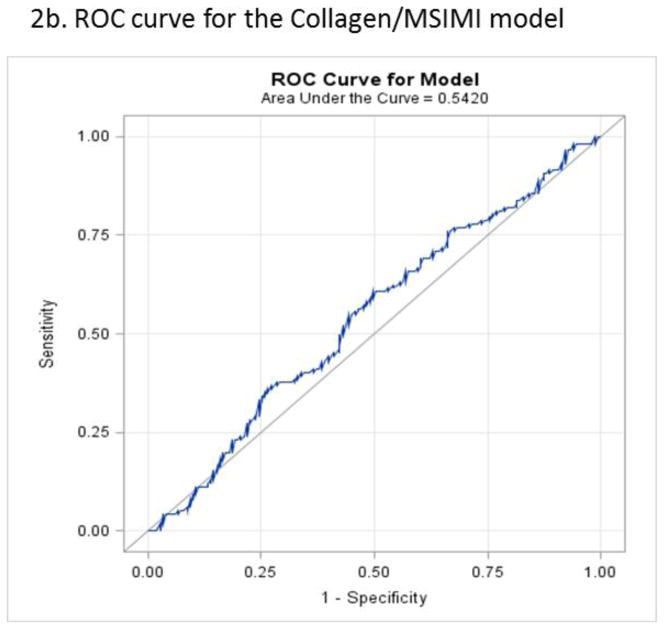

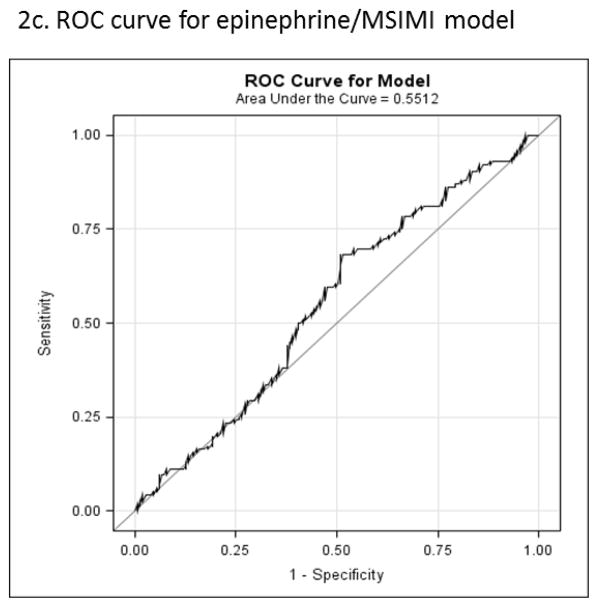

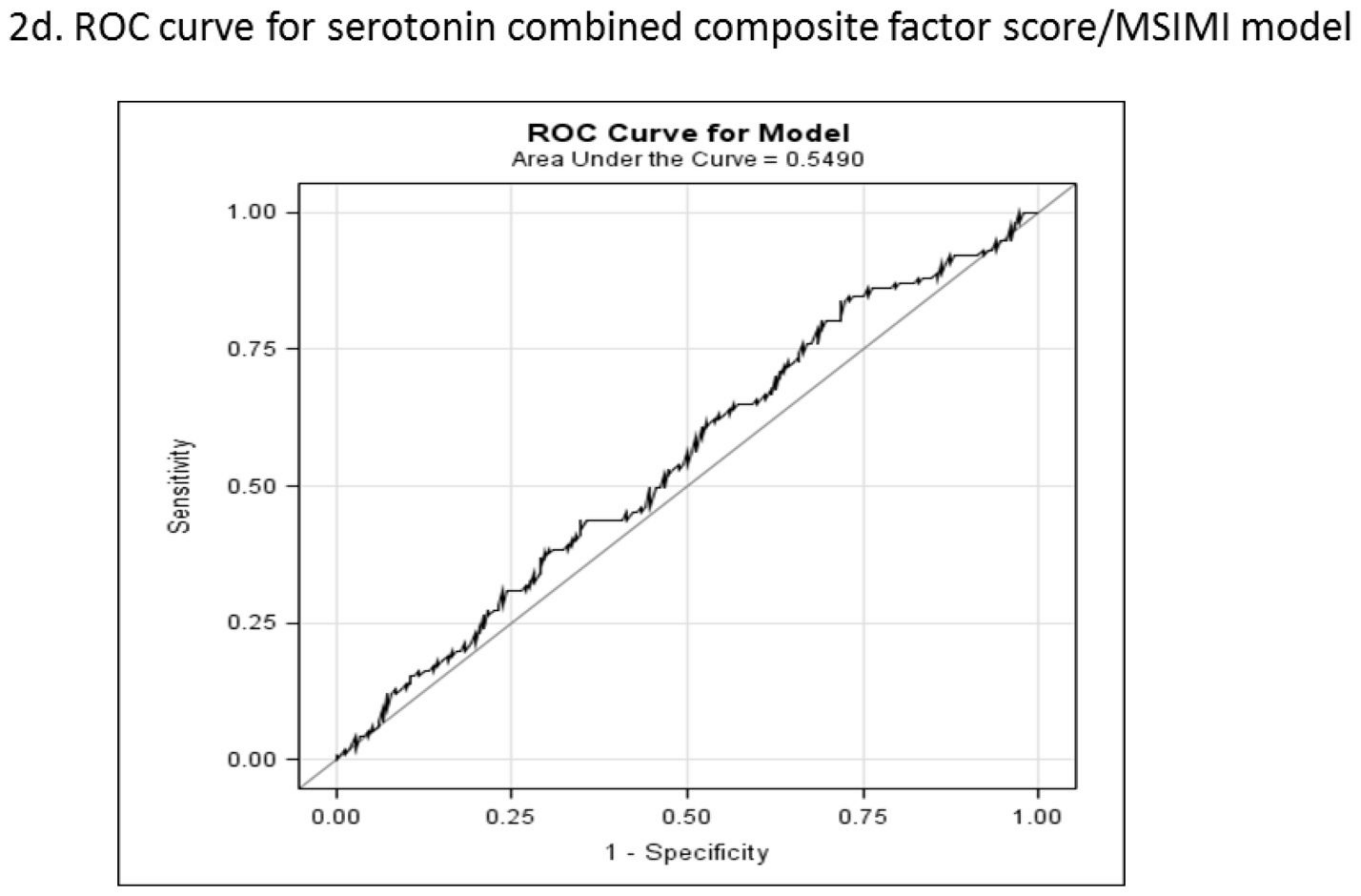

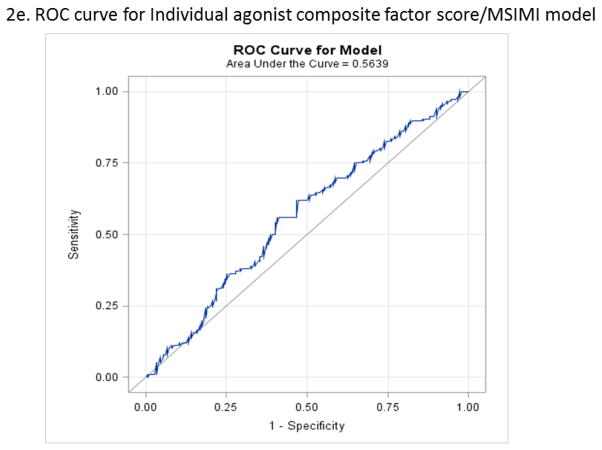
Figure 2a. Area under the receiver operating characteristic curve for serotonin plus ADP stimulated stress-induced platelet reactivity predicting MSIMI. Mental stress-induced platelet aggregation scores are adjusted for age, sex, smoking status, BDI-scores, and resting platelet aggregation. Area under the curve=.59 Figure 2b. Area under the receiver operating characteristic curve for Collagen 10uM stimulated stress-induced platelet reactivity predicting MSIMI. Mental stress-induced platelet aggregation scores are adjusted for age, sex, smoking status, BDI-scores, and resting platelet aggregation. Area under the curve=.54 Figure 2c Area under the receiver operating characteristic curve for Epinephrine 10uM stimulated stress-induced platelet reactivity predicting MSIMI. Mental stress-induced platelet aggregation scores are adjusted for age, sex, smoking status, BDI-scores, and resting platelet aggregation. Area under the curve=.55 Figure 2d. Area under the receiver operating characteristic curve for composite score of serotonin plus individual agonist stimulated stress-induced platelet reactivity predicting MSIMI. Mental stress-induced platelet aggregation scores are adjusted for age, sex, smoking status, BDI-scores, and resting platelet aggregation. Area under the curve=.55 Figure 2e. Area under the receiver operating characteristic curve for composite score of individual agonist stimulated stress-induced platelet reactivity predicting MSIMI. Mental stress-induced platelet aggregation scores are adjusted for age, sex, smoking status, BDI-scores, and resting platelet aggregation. Area under the curve=.56

Analysis of the ROC curve in the entire sample found that neither the covariate adjusted serotonin + ADP (AUC = 59), epinephrine (AUC = 55), nor collagen (AUC = 54) scores were good discriminators of MSIMI status (Figure 2a and b). The individual agonist (AUC = 56) and combined agonist (AUC = 55) composite scores were also not good discriminators of MSIMI (Figure 2c and d). Examination of these curves also shows that there is not a single cut-off value that best discriminates between patients exhibiting MSIMI and those who do not.

## DISCUSSION

Platelet hyperactivity is a significant element of the complex multifactorial pathophysiological process of IHD (31). Previous studies have demonstrated that acute episodes of anger or severe emotional stress may trigger acute MI and other forms of ACS (31), and mental stress may enhance platelet activation in patients with angina and IHD (9,12,13,32). The present study potentially expands those findings by demonstrating that mental stress-induced platelet hyper-aggregation occurred in patients who developed MSIMI but not in patients whose left ventricle responded normally to mental stress testing. Our analysis showed significant relationships between the mental stress-induced platelet aggregation with the mental stress-induced WMSI and LVEF changes, i.e. the higher the platelet aggregation, the greater the WMSI elevation and the LVEF reduction caused by mental stress testing. These results elucidate platelet hyper-aggregation in response to emotional stress as a potential mechanism underlying MSIMI.

The complexity and dynamics of platelet aggregation as well as its interaction with other elements of the complex multifactorial pathophysiological process of IHD, such as inflammation, microvascular coronary dysfunction, endothelial dysfunction, and angiogenesis, was not unraveled until recently(33–36). Our analysis showed that patients with MSIMI in contrast to those with NLVR, experienced mental stress-induced, but not resting, platelet hyper-aggregation. This pattern of association supports a role for mental stress-induced heightened platelet aggregation in MSIMI. In contrast to the prior observations from our group in healthy individuals not receiving platelet antagonists, resting platelet aggregation in response to epinephrine was not bimodal and there were relatively few patients whose aggregation was above 60% (19). Usage of aspirin (> 95% of patients) and taking PPRB (42.1% of patients) might explain the findings. Usage of these medications was evenly distributed among the groups and had little effect on the associations between mental stress-induced platelet aggregation and MSIMI. One can speculate, then, that the mechanisms underlying MSIMI related platelet aggregation are independent of the inhibitory effects of aspirin and the PPRB on platelet aggregation. Approximately 10% of patients with ACS experience recurrent thrombotic events or death even with current antiplatelet treatment (37,38).

Our findings suggest that the mental stress strategy employed in the present study in patients with known coronary artery disease evoked an objective ischemic response that is associated with platelet hyper-aggregation to the mental stress test, and that serotonin, ADP, and collagen may play significant contributing roles in the mental stress-induced platelet hyper-aggregation (19,39). Discovery (40,41) and increased understanding of the clinical significance of MSIMI (2,3,42) has become a hallmark advance in the search for pathophysiological-mechanisms whereby psychosocial factors contribute to adverse cardiovascular outcomes (43–46). These advances underscore the need to shift from simple epidemiological studies documenting the impact of mental stress on human health to more a personalized focus on identifying susceptible individuals whose neurological and cardiovascular systems respond adversely to acute psychosocial stress. The present study specifically identifies a pathophysiological mechanism, i.e., increased platelet aggregation to acute mental stress underlies MSIMI that occurs in a vulnerable subpopulation of IHD patients. The findings suggest that application of mental stress testing may be imperative to identify a subpopulation of IHD patients who are at high risk for poor prognosis. We speculate that mental stress-induced platelet hyper-aggregation is an underlying pathological mechanism of MSIMI. However, it is also possible that other pathological processes underlying MSIMI trigger platelet hyper-aggregation despite aspirin and PPRB usage. Considering either scenario, one must acknowledge that a combination of coronary artery disease, heightened platelet aggregation to emotional stress, and MSIMI is concerning. Studies have indicated that the onset of acute MI and other forms of ACS triggered by acute emotional distress tended to happen within a critical time window of about 2 h (31). The study of MSIMI potentially provides a model for better understanding the role of emotional stress in triggering ACS in susceptible individuals.

The reasons for lack of associations observed between the psychological measures with platelet aggregations (rest and after mental stress) and MSIMI status remain unknown. The reasons for the lack of associations observed between the psychological measures and MSIMI status remain unknown. There are a number of factors that could be speculated to explain such findings. One could be that the subjective feelings to emotional stress may not be fully formed and well recognized during stressors of short duration, such as those used in the current study. Other factors such as denial, minimization, or socially desirable responding may result in underreporting of emotional distress thereby obfuscating possible associations between MSIMI and self-reported emotional responses. Alternatively, ratings of the affect collected during the mental stress protocol might be an accurate representation of the emotional responses experienced by the NLVR and MSIMI groups, but cardiovascular profiles during mental stress were vastly different between these two groups. This suggests that MSIMI may not simply a function of a mental propensity to experience more intense periods of mental stress, but may reflect other factors both in the central nervous system and/or the periphery (e.g. greater prevalence of small vessel disease). Choosing among these explanations is beyond the scope of the current study.

The unique aspect of the present study is connecting mental stress-induced platelet hyper-aggregation with MSIMI. Psychophysiological stress testing has been used to study the impact of acute stress on platelet activation (47). However, few studies have tested cardiac patients and methods of measuring the platelet responses have varied, leading, not surprisingly, to mixed results. For instance, one study showed plasma thromboglobulin higher during mental stress in patients with stable angina than healthy controls (13), but another study showed healthy men had a greater stress-induced rise in thromboglobulin than post-MI patients (12). Variations in measurement methods, timing of platelet responses, and use of antiplatelet medication are also critical components when evaluating stress-induced platelet activities (42). Our study extends those experiments by identifying a subset of patients IHD patients who appear to be particularly prone to hyper-aggregation during mental stress. The duration of the mental tasks applied in our study resulted in significant variability in platelet aggregation changes with some patients exhibiting increases in platelet aggregation and others showing significant decreases in platelet aggregation. The fact that the platelet aggregation after mental stress testing was well separated from the resting platelet aggregation suggested that the mental test protocol altered the internal environment or hemostatic system of these patients. One limitation to this design is that we cannot discern the independent effects of each mental task on platelet aggregation change and how long and the duration of the mental stress induced platelet aggregation.

It is worth noting that our analysis revealed significant relationships between mental stress-induced platelet aggregation and mental stress-induced WMSI and LVEF changes, though the magnitude of those associations were not particularly strong. Mental stress induced LVEF reduction is associated with increased adverse cardiovascular events in IHD patients (3,4). The significant associations with mental stress-induced LVEF reduction and platelet hyper-aggregation may be a cause and/or contributor to global left ventricular dysfunction. The lack of a well delineated cut-off value for mental stress-induced platelet hyper-aggregation measurements to predict MSIMI with high sensitivity and specificity may reflect the role of additional pathological processes in MSIMI, including elevated peripheral vascular resistance, endothelial dysfunction (48,49) and inflammatory processes (50) that are all cardinal pathological processes underlying IHD and other cardiovascular diseases (51,52). Mental stress testing has been found to induce transient vasoconstriction in non-significantly stenosed coronary arteries (53,54) and impair flow mediated dilation of peripheral arteries (55–57). Because no previous experiment has measured these parameters comprehensively and technical limitations make it difficult to assess the temporal relationships among these pathological processes, we lack evidence about whether these changes occur in sequence or simultaneously. Other potential mechanisms, such as insufficient energy production or mitochondrial dysfunction (58–60), may also be contributory mechanism. Negative appraisal of the mental tasks used in this study by central nervous system processes may be the initial step in the cascade of the pathological process resulting in MSIMI.

## CONCLUSION

In conclusion, the study demonstrated that mental stress-induced platelet hyper-aggregation is associated with MSIMI occurrence. The dynamic change of stress-induced platelet aggregation may be a cause of MISMI, and/or consequence of other pathological process of MSIMI. The novel findings call out to a concerning pathological mechanism that is previously unknown and potentially hazardous for patients with IHD. Studies are needed to better understand the mechanisms of how mental stress resulted in dynamic alternation of hemostasis. Interventions that attenuate the stress-induced platelet hyper-aggregation and modify MSIMI merit attention in future studies.

## Figures and Tables

**Table 1:** Baseline Demographic and Clinical Characteristics

Variable	NLVR N=49 %	MSIMI N=117 %	Intermediate N=104 %	P-value*
**Demographics**				
**Age, mean (SD), years**	61.18 (9.36)	63.39 (10.86)	63.77 (10.22)	0.22
**Gender (Female)**	2 (4.08)	23 (19.66)	13 (12.50)	0.011
**Race (Non-White)**	5 (10.20)	25 (21.37)	14 (13.46)	0.088
**Medical History**				
**Diabetes**	16 (32.65)	36 (30.77)	27 (25.96)	0.81
**Current Angina**	10 (20.41)	24 (20.51)	19 (18.27)	0.95
**Prior Myocardial Infarction**	15 (30.61)	59 (50.43)	49 (47.12)	0.064
**Prior PCTA**	28(57.14)	75 (64.10)	67 (64.42)	0.40
**Prior CABG**	22(44.90)	55 (47.01)	44 (42.31)	0.80
**Congestive heart Failure**	1 (2.04)	7 (5.98)	9 (8.65)	0.28
**Hypertension**	38 (77.55)	91 (77.78)	87 (83.65)	0.98
**Hyperlipidemia**	47 (95.92)	109 (93.16)	99 (95.19)	0.50
**Current Tobacco Use**	4 (8.16)	20 (17.09)	12 (11.54)	0.28
**Depression**	5 (10.20)	18 (15.38)	14 (13.46)	0.38
**Medications**				
**Aspirin**	47 (95.92)	113 (97.41)	98 (95.15)	0.61
**Additional Antiplatelet Drugs**	20 (40.82)	54 (46.55)	40 (38.46)	0.50
**ACE-I/ARB**	38(77.55)	94(81.03)	76(73.08)	.61
**Calcium Channel Blocker**	9 (18.75)	24 (20.69)	28 (26.92)	0.78
**Beta blocker**	38 (77.55)	100 (86.21)	88 (84.62)	0.17
**Statin**	46 (93.88)	109 (94.78)	92 (89.32)	0.82
**Psychological Measures**				
**BDI**	8.46 (7.92)	9.01 (7.03)	7.93 (6.89)	0.66
**CES-D**	28.96 (7.66)	31.02 (9.02)	29.83 (7.92)	0.16
**STAI-Trait**	33.69(11.18)	34.62(10.83)	33.64(9.84)	0.62
**STAI-State**	27.63 (8.26)	29.70 (10.45)	28.33 (7.66)	0.22
**PSS**	22.15 (7.28)	23.28 (7.49)	22.74 (6.72)	0.38

* The P values reflect t-tests (continuous variables) and chi-squares (categorical variables) assessing bivariate relations between demographic/clinical characteristics and MSIMI status (MSIMI/NLVR).

** Approximately 15% (N = 40) of the patients had received prior PTCA and CABG.

Abbreviations: ACE-I, angiotensin-converting enzyme inhibitor; ARB, angiotensin receptor blocker; CABG, coronary artery bypass grafting; PTCA, percutaneous coronary angiography; BDI, Beck Depression Inventory; CESD, Center for Epidemiologic Studies Depression Scale; STAI-Trait, Spielberger Trait anxiety Scale; STAI-State, Spielberger State anxiety Scale; PSS, Perceived stress scale.

BDI, score range, 0 to 63 (higher score=greater severity of depressive symptoms).

CESD, score range, 0 – 60 (higher score=greater severity of depressive symptoms).

PSS, Score range, 10 to 50 (higher score=greater levels of perceived stress).

Spielberger State-Trait Anxiety Inventory scales. Score ranges: trait anxiety, 20 to 80 (higher score=greater levels of trait anxiety); state anxiety, 20 to 80 (higher score=greater levels of state anxiety.

**Table 2:** Resting Bio-Psychological Measurements in Laboratory

Variable	NLVR N=49	MSIMI N=117	Intermediate N=104	P-value*
**Variables Summarized in Mean (SD)**				
**Resting CV**				
**Systolic blood pressure**	126.06(17.00)	125.88 (17.17)	125.37 (19.45)	0.95
**Diastolic blood pressure**	74.73 (12.28)	71.54 (11.31)	71.96 (11.48)	0.12
**Heart rate**	68.93 (10.11)	67.54 (11.21)	66.32 (10.37)	0.47
**Left ventricular ejection fraction**	55.51 (8.73)	55.27 (11.85)	59.39 (8.40)	0.90
**Wall motion score index**	1.15 (0.35)	1.29 (0.43)	1.15 (0.33)	0.044
Emotional State**				
**Sadness**	6.13(15.42)	9.16(17.72)	6.86(15.20)	.30
**Tension**	14.56(20.94)	19.10(22.75)	15.44(18.58)	.24
**Frustration**	7.15(15.97)	10.71(21.15)	6.16(13.54)	.30
**Calm**	83.79(24.30)	81.94(22.33)	86.57(16.09)	.64
**In Control**	80.31(27.61)	80.05(24.83)	84.24(20.09)	.95
***Variables Summarized in Mean (SE)				
**Serotonin Transporter Expression**				
**Bmax**	205.75 (25.14)	235.94 (16.65)	208.35 (10.08)	0.33
**Kd100**	375.16 (26.36)	357.15 (17.45)	363.48 (19.90)	0.58
**Vmax**	142.61 (8.92)	161.17 (5.72)	148.31 (13.79)	0.085
**Platelet Aggregation (AUC)**				
**ADP 5 μM**	239.83 (16.73)	241.64 (10.60)	257.69 (10.98)	0.93
**Epinephrine 10 μM**	156.69 (16.29)	140.29 (10.32)	143.95 (9.31)	0.40
**Collagen 10 μM**	274.81 (14.21)	280.76 (9.00)	270.20 (10.48)	0.73
**Serotonin+ADP**	217.43 (15.78)	209.36 (10.00)	227.78 (9.73)	0.70
**Serotonin+Epinephrine**	249.72 (13.80)	239.72 (8.75)	249.43 (8.33)	0.55
**Serotonin+Collagen**	231.48 (13.98)	239.79(8.86)	246.54 (8.62)	0.62

* The P-values for resting CV and state emotion variables were estimated with ANOVAs comparing MSIMI (N = 117) and NLVR (N = 49) groups.

** State affects were measured by visual analog scales (0–100; higher score=greater levels of state negative or positive affect).

*** The P values for platelet variables reflect ANCOVAs comparing variables between MSIMI (N = 117) and NLVR (N = 48) groups controlling for sex, age, smoking status, BDI scores, and resting LVEF.

**Table 3:** Mental Stress Induced Bio-Psychological Reactivity and Platelet Aggregation (Post-stress – Resting)

Variables	NLVR N=48	MSIMI N=117	Intermediate N=104	P-value*
**Variables Summarized as Mean (SE)**				
**Cardiovascular Reactivity**				
**Δ Systolic blood pressure**	29.88(1.87)	26.52(1.19)	24.35(1.21)	.14
**Δ Diastolic blood pressure**	15.25(1.35)	14.59(0.86)	12.95(0.83)	.69
**Δ Heart rate**	10.94(1.31)	9.28(0.84)	8.57(0.78)	.29
**Δ Wall motion score index**	−0.01(0.02)	0.11(0.01)	−.003(.002)	<.001
**Δ Left ventricular ejection fraction**	4.65(0.63)	−2.54(0.42)	−0.93(0.24)	<.001
**Δ Platelet Aggregation (AUC)**				
**ADP 5 μM**	−2.47(7.92)	9.55(4.97)	−2.55(6.47)	.21
**Epinephrine, 10 μM**	−6.40(7.61)	12.84(4.84)	11.04(7.74)	.037
**Collagen, 10 μM**	−14.23(8.75)	6.95(5.54)	6.98(7.10)	.045
**Individual Agonist Composite Score**	−0.27(0.14)	0.10(0.09)	−0.01(0.12)	.030
**Serotonin+ ADP**	−27.24(8.34)	6.64(5.29)	−9.19(5.66)	<.001
**Serotonin+Epinephrine**	−11.58(9.31)	2.57(5.90)	7.43(6.56)	.21
**Serotonin+Collagen**	−45.10(9.90)	−31.36.(6.27)	−36.33(6.46)	.25
**Serotonin + Agonist Composite Score**	−0.29(0.15)	0.12(0.09)	0.02(0.10)	.019
**Emotional Response**				
**Sadness**	7.90(2.67)	7.61(1.68)	11.21(2.01)	.93
**Tension**	28.22(3.12)	23.10(1.97)	27.78(2.49)	.17
**Frustration**	44.93(3.17)	42.07(2.00)	44.98(2.64)	.45
**Calm**	−28.59(3.24)	−24.95(2.04)	−29.24(2.54)	.35
**In Control**	−31.17(2.98)	−27.11(1.88)	−29.22(2.75)	.26

* The P values reflect ANCOVAs comparing variables between MSIMI (N = 117) and NLVR (N = 48) groups controlling for age, sex, smoking status, BDI scores, resting LVEF, and appropriate resting cardiovascular index.

**Table 4:** Multivariate logistic regression of platelet aggregation variables predicting MSIMI (MSIMI/NLVR) status

Variables	Odds Ratio(95%CI)*	P-value
**Δ Platelet Aggregation**		
**ADP, 5 μM**	1.005(0.998 – 1.013)	.19
**Epinephrine, 10 μM**	1.009(1.000 – 1.017)	.054
**Collagen, 10 μM**	1.006(1.000 – 1.012)	.053
**Individual Agonist Composite Score**	1.57(1.05 – 2.36)	.028
**Serotonin+ ADP**	1.012(1.005–1.02)	.001
**Serotonin+Epinephrine**	1.003(0.998 – 1.009)	.25
**Serotonin+Collagen**	1.003(0.998 – 1.009)	.22
**Serotonin + Agonist Composite Score**	1.59(1.07 – 2.37)	.022
**Resting Platelet Aggregation**		
**ADP, 5 μM**	1.000(0.997 – 1.004)	.80
**Epinephrine, 10 μM**	1.000(0.996 – 1.004)	.94
**Collagen, 10 μM**	1.002(0.998 – 1.006)	.36
**Serotonin+ ADP**	1.000(0.997 – 1.004)	.93
**Serotonin+Epinephrine**	0.999(0.995 – 1.003)	.77
**Serotonin+Collagen**	1.002(0.998 – 1.006)	.37

* Odds ratios reflect the change in the odds of having MSIMI associated with an increase of one unit in the platelet aggregation variables.

** Models included age, sex, smoking status, BDI-scores and resting LVEF.

## Abbreviations

MSIMI: Mental Stress Induced Myocardial Ischemia
MI: Myocardial Ischemia
ACS: Acute Coronary Syndrome
IHD: Ischemic Heart Disease
REMIT: Responses of Myocardial Ischemia to Escitalopram Treatment
NLVR: Normal Left Ventricular Response
ECG: Electrocardiography
LVEF: Left Ventricular Ejection Fraction
PPRB: Platelet P2Y_12_ Receptor Blocker
PRP: Platelet-rich Plasma
PPP: Platelet-poor Plasma
LTA: Light Transmittance Aggregometry
WMSI: Wall Motion Score Index
ROC: Receiver-operating Characteristic (curves)

## Appendix 1

### Mental Stress Testing Protocol

The three mental stress tasks, i.e. Mental arithmetic, Public speaking with anger recall, and Mirrior tracing, used in REMIT have been used by many investigators to elicit myocardial ischemia in laboratory (Strike 2003). Depending on purposes of individual studies, choices of mental stress tasks; the number, kind, as well as the duration of tasks, had varied among studies. We chose these three mental tasks for the REMIT primarily based on our experiences gathered from the MIIT (Myocardial Ischemia Intervention Trial) study (Jiang & Blumenthal Direction in psychiatry 2001; 21:9–21). The MIIT study used 5 mental tasks, i.e., mental arithmetic, mirror tracing, speech with anger-recall, general material reading, and Type A personality interview. The results of MIIT revealed that the three tasks, mental arithmetic, mirror tracing, speech with anger-recall, were able to elicit almost all the ischemic activities. Compared to the Type A personality interview task, these three tasks were less influenced by tester variability. General material reading was used as a reference task. Therefore we chose these 3 mental tasks for REMIT study. For the purpose of REMIT study, the three tasks were delivered in the fixed sequence.

1. Mental arithmetic: patients were asked to perform a series of serial subtractions beginning at a given number which were different for each repeated test and chosen by the tester from a fixed list of various numbers, with encouragement to perform calculations as quickly and accurately as possible;
2. Public speaking with anger recall: patients were asked to give a brief speech on a recent situation in which they experienced anger or upset to an audience of 2–3 observers after 1 minute of preparation. Participants were instructed to have the speech well organized with clearly defined issue and concise content, as well as the speech would be evaluated. If they run out of what to say, the research staff would prompt them with questions to elicit more content until the 3-minute was up;
3. Mirror trace: patients were asked to outline, as quickly and accurately as possible, a star from its reflection in a mirror. The device of the mirror trace beeps and records errors participants made during the testing.

Each task lasted 3 minutes along with a least of a 6-minute rest period between tasks.
